# Tumor necrosis factor-like weak inducer of apoptosis (TWEAK) promotes glioma cell invasion through induction of NF-κB-inducing kinase (NIK) and noncanonical NF-κB signaling

**DOI:** 10.1186/s12943-014-0273-1

**Published:** 2015-01-27

**Authors:** Evan M Cherry, Dong W Lee, Ji-Ung Jung, Raquel Sitcheran

**Affiliations:** Department of Molecular and Cellular Medicine, Texas A&M University College of Medicine, College Station, TX USA; Medical Science Graduate 588 Program, Texas A&M University College of Medicine, College Station, TX USA; The Texas Brain and Spine Institute, Bryan, TX USA

**Keywords:** Glioma, Glioblastoma, Noncanonical NF-κB, RelB, TWEAK, MMP, NIK, Invasion

## Abstract

**Background:**

High-grade gliomas are one of the most invasive and therapy-resistant cancers. We have recently shown that noncanonical NF-κB/RelB signaling is a potent driver of tumorigenesis and invasion in the aggressive, mesenchymal subtype of glioma. However, the relevant signals that induce activation of noncanonical NF-κB signaling in glioma and its function relative to the canonical NF-κB pathway remain elusive.

**Methods:**

The ability of tumor necrosis factor (TNF)-like weak inducer of apoptosis (TWEAK) to regulate NF-κB signaling and promote tumor progression was investigated in both established and primary high-grade glioma tumor lines using a three-dimensional (3-D) collagen invasion assay. The roles of specific NF-κB proteins in regulating glioma cell invasion and expression of Matrix Metalloproteinase 9 (MMP9) in response to TWEAK were evaluated using shRNA-mediated loss-of-function studies. The ability of NF-κB-inducing kinase (NIK) to promote glioma growth *in vivo* was investigated using an orthotopic xenograft mouse model.

**Results:**

In glioma cells that display elevated noncanonical NF-κB signaling, loss of RelB attenuates invasion without affecting RelA expression or phosphorylation and RelB is sufficient to promote invasion in the absence of RelA. The cytokine TWEAK preferentially activates the noncanonical NF-κB pathway through induction of p100 processing to p52 and nuclear accumulation of both RelB and p52 without activating the canonical NF-κB pathway. Moreover, TWEAK, but not TNFα, significantly increases NIK mRNA levels. TWEAK also promotes noncanonical NFκB-dependent MMP9 expression and glioma cell invasion. Finally, expression of NIK is sufficient to increase gliomagenesis *in vivo*.

**Conclusions:**

Our data establish a key role for NIK and noncanonical NF-κB in mediating TWEAK-induced, MMP-dependent glioma cell invasion. The findings also demonstrate that TWEAK induces noncanonical NF-κB signaling and signal-specific regulation of NIK mRNA expression. Together, these studies reveal the important role of noncanonical NF-κB signaling in regulating glioma invasiveness and highlight the therapeutic potential of targeting activation of NIK in this deadly disease.

**Electronic supplementary material:**

The online version of this article (doi:10.1186/s12943-014-0273-1) contains supplementary material, which is available to authorized users.

## Background

The aggressive, infiltrative growth of gliomas renders them highly refractory to surgery, radiation and chemotherapy. Thus, understanding the biochemical and molecular pathways that control glioma cell invasion is critical for identifying more effective therapeutic targets. Aberrant activation of the canonical NF-κB pathway is well documented in a variety of malignancies [1,2] and plays important roles in regulating glioma invasion and tumor progression [3-5]. However, specific roles for the noncanonical NF-κB pathway in tumor pathogenesis, particularly in solid tumors of the central nervous system (CNS), are poorly understood.

NF-κB proteins are evolutionarily conserved transcription factors that play central roles as coordinators of key biological processes, including immunity, inflammation, cell death and survival. The five mammalian family members RelA (p65), RelB, c-Rel, NFKB1 (p105/p50), and NFKB2 (p100/p52) share an evolutionarily conserved Rel homology domain that mediates DNA binding and dimerization with other NF-κB subunits. NF-κB complexes are held in the cytoplasm in an inactive state, bound to members of the NF-κB inhibitor (IκB) family, which includes IκBα. In response to specific extracellular signals such as the cytokine tumor necrosis factor-α (TNFα), activation of canonical NF-κB signaling is triggered by signal-induced phosphorylation and degradation of IκBα, followed by nuclear translocation of the active, liberated RelA-p50 complexes. IκBα phosphorylation-induced degradation and activation of the canonical NF-κB pathway is dependent on IκB kinase-β (IKKβ).

Noncanonical NF-κB signaling is mediated by RelB-p52 heterodimers whose activation is dependent on NF-κB-inducing kinase (NIK). NIK, a mitogen-activated protein kinase kinase kinase (MAP3K14), is regulated primarily through protein stabilization [6]. In unstimulated cells, NIK interacts with TNF receptor associated factor-3 (TRAF3) in a multi-subunit E3 ubiquitin ligase complex which leads to NIK polyubiquitination and proteasomal degradation [7]. Consequently, NIK protein levels are maintained at low levels. A critical step in activation of noncanonical NF-κB signaling is the ubiquitination-mediated proteasomal degradation of TRAF3, which stabilizes NIK protein, leading to IKKα activation and phosphorylation of p100, an inhibitory IκB-like protein that retains RelB in the cytoplasm. Phosphorylation of p100 leads to its proteolytic processing to form p52, culminating in nuclear translocation of transcriptionally active RelB-p52 heterodimers [8,9]. Notably, unlike other NF-κB proteins, RelB is inherently unstable and its protein levels are stabilized by interaction with p100/p52 in the cytoplasm [10] and DNA binding in the nucleus [11].

The noncanonical NF-κB pathway can be specifically activated by signals such as B-cell-activating factor receptor (BAFFR) [6]. Some signals, such as TNF-like weak inducer of apoptosis (TWEAK) have been shown to regulate both the canonical and noncanonical NF-κB pathways for sustained NF-κB activation [12]. TWEAK and its receptor, fibroblast growth factor-inducible protein 14 (Fn14), have recently been implicated in tumor cell pathogenesis [13,14] and upregulation of MMP9 [15], which is generally associated with poor disease prognosis due to its ability to promote tumor growth, migration, invasion, and metastasis [16]. However, the functional outcomes of TWEAK signaling in glioma with regard to specific downstream NF-κB signaling events are not clear. We have previously demonstrated that the noncanonical NF-κB protein, RelB, is highly expressed in a subset of aggressive, mesenchymal glioma, where it is a potent driver of oncogenesis and a predictor of survival in human patients [17]. In this study, we investigate the effects of TWEAK on noncanonical NF-κB/RelB signaling, MMP9 expression and glioma invasion.

## Results

### The invasive potential of glioma tumor lines positively correlates with RelB expression

**Figure 1:**
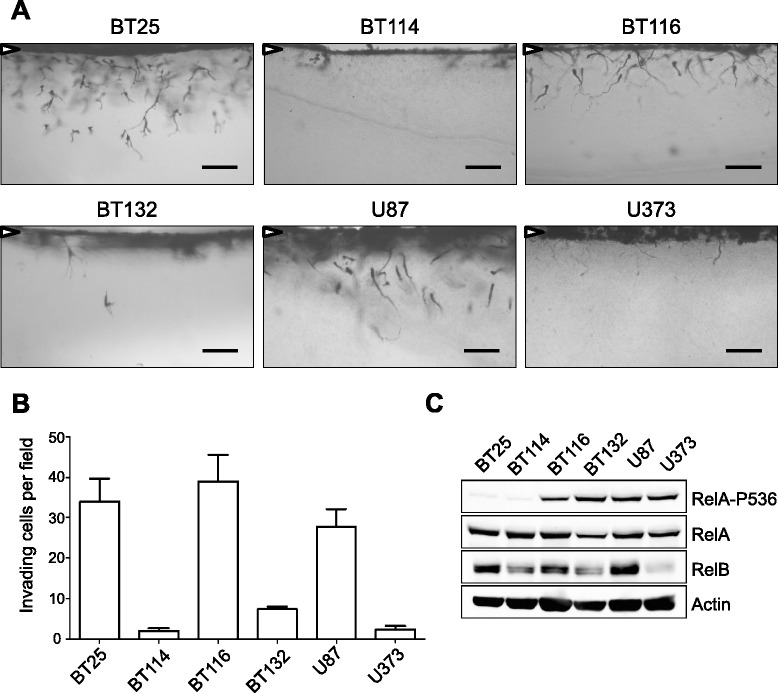
**Glioma cell invasion correlates positively with RelB expression.** Equal numbers of glioma cells from indicated cell lines were seeded on 3-D collagen matrices and allowed to invade for 48 h, fixed with 3% glutaraldehyde, and stained with 0.1% toluidine blue. **(A)** Side-view light microscopy images were taken from thin slices of the collagen matrices at 10x magnification. White arrowheads indicate cell monolayer. Scale bars = 100 μm. **(B)** Quantification of invasion density for cell lines in Figure 1A. Data represent average numbers of invading cells per 1-mm^2^ field (n = 3 wells) ± S.E.M. **(C)** Western blot of whole-cell lysates from glioma lines using the indicated antibodies.

Because aggressive invasion of high-grade gliomas into normal health tissue is a significant barrier to treatment, we sought to further evaluate the relationship between NF-κB signaling pathways and glioma cell invasion. First, we analyzed the invasive potential of both established high-grade glioma tumor lines (U87 & U373) and primary brain tumor-derived lines (BT25, BT114, BT116, BT132) using a three-dimmensional (3-D) collagen matrix assay [18]. Compared with two-dimensional (2-D) assays, modeling tumor cell invasion in 3-D collagen matrices better reflects both the *in vivo* cell-cell and cell-extracellular matrix interactions within a tumor [19], as well as the significantly elevated levels of collagen in the adult brain tumor microenvironment [20]. Analysis of side-view images of cells invading the collagen matrix and quantification of invading cells demonstrated that BT25, BT116, and U87 cells were the most invasive tumor lines, while BT132, BT114 and U373 were significantly less invasive (Figure 1A). Quantification of invasion density confirmed these observations (Figure 1B).

Next, we analyzed expression of canonical and noncanonical NF-κB proteins in the different glioma tumor lines. Western blot analysis demonstrated that, compared with BT25 and BT114 cells, BT116, BT132, U87 and U373 cells exhibited higher levels of phosphorylated RelA at serine 536 (RelA-P536), an marker of enhanced NF-κB transcriptional activation potential [21,22], while total RelA levels were comparable in all cell lines (Figure 1C). We noted that neither total RelA, nor RelA-P536 levels, correlated with invasiveness. For example, low RelA-P536 is seen in both invasive BT25 cells, as well as low-invasive BT114 cells. Likewise, high RelA-P536 expression was observed in both high-invasive BT116 and low-invasive BT132 (see Figure 1A, B). In contrast to RelA, RelB protein levels were more varied in the different glioma lines, and high RelB-expressing cells (U87, BT25, and BT116) invaded much more efficiently than low RelB-expressing cells (BT132, BT114 and U373). These results demonstrate that levels of RelB, but not RelA or RelA-P536, strongly correlate with the invasive potential of glioma cells.

### RelB poteniates glioma invasion independently of RelA

**Figure 2:**
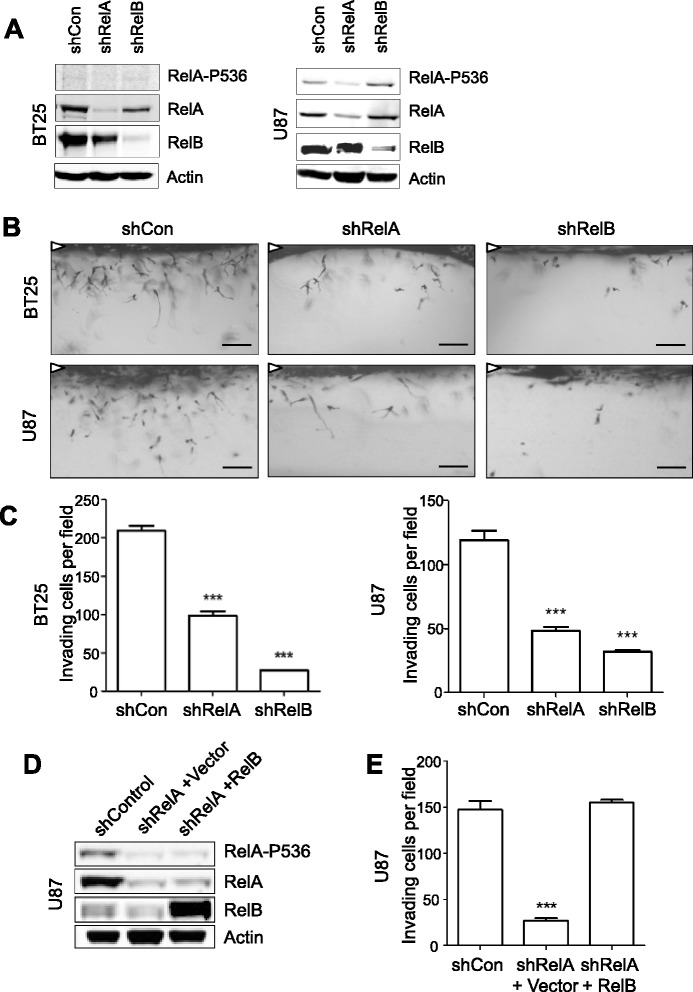
**RelB poteniates glioma invasion independently of RelA. (A)** Western blot of whole-cell lysates from NSC-cultured BT25 and U87 cells transduced with lentiviral vectors expressing shRNA targeting RelA (shRelA), RelB (shRelB), or a scrambled control (shCon)*.* **(B)** Representative images of invading shRNA-transduced glioma cells at 48 h. White arrowheads indicate monolayer. Scale bars = 100 μm. **(C)** Quantification of invasion density at 48 h for BT25 and U87 knockdown cell lines. Data represent average numbers of invading cells per 1-mm^2^ field (n = 3 wells) ± S.E.M. ***p < 0.001 relative to shCon using One-way ANOVA with Tukey’s H.S.D. post-test. **(D)** Western blot of whole-cell lysates from U87 shRelA cells transduced with lentiviral vectors containing RFP cDNA (+Vector) or RelB cDNA (+RelB). **(E)** Quantification of invasion density at 48 h for U87 shRelA cells in Figure 2D. ***p < 0.001 relative to shCon using One-way ANOVA with Tukey’s H.S.D. post-test.

We have previously reported that loss of RelB in glioma significantly reduces the number and depth of invading tumor cells [17]. To further characterize and compare the roles of RelA and RelB in invasion, we used lentivirally-delivered shRNA to inhbit expression of these proteins in the highly invasive BT25 and U87 cells (Figure 2A). Side-view images of 3-D collagen invasion matrices and quantification of invading cells demonstrated that loss of either RelA or RelB each resulted in a significant decrease of glioma invasive potential (Figure 2B, C and Additional file 1: Figure S1A, B). In shRelA cells, a significant loss of RelB was observed (Figure 2A), consistent with the known ability of RelA to regulate expression of RelB [23]. In contrast, RelA protein levels were not significantly affected in shRelB cells (Figure 2A), demonstrating that the diminished invasive potential was not due to loss of signaling through the canonical, RelA-dependent, NF-κB pathway.

Next, we tested whether ectopic expression of RelB could restore the invasive potential of RelA knockdown cells. U87 shRelA cells were transduced with lentivirus to express either a control vector or RelB. We observed that ectopic expression of RelB in the RelA knockdown cells was approximately 5-fold higher than endogenous RelB expression in shControl cells (Figure 2D and Additional file 1: Figure S1C). Notably, overexpression of RelB did not affect shRNA-mediated knockdown of RelA and also did not alter the levels of either total or phosphorylated RelA (Figure 2D). RelB was nevertheless able to restore the loss of invasion observed in RelA knockdown cells (Figure 2E). These results demonstrate that in glioma cells with attenuated levels of RelA protein, RelB can rescue invasion without increasing the expression or activity of RelA and canonical NF-κB signaling.

### TWEAK promotes glioma cell invasion and predominantly activates noncanonical NF-κB signaling

**Figure 3:**
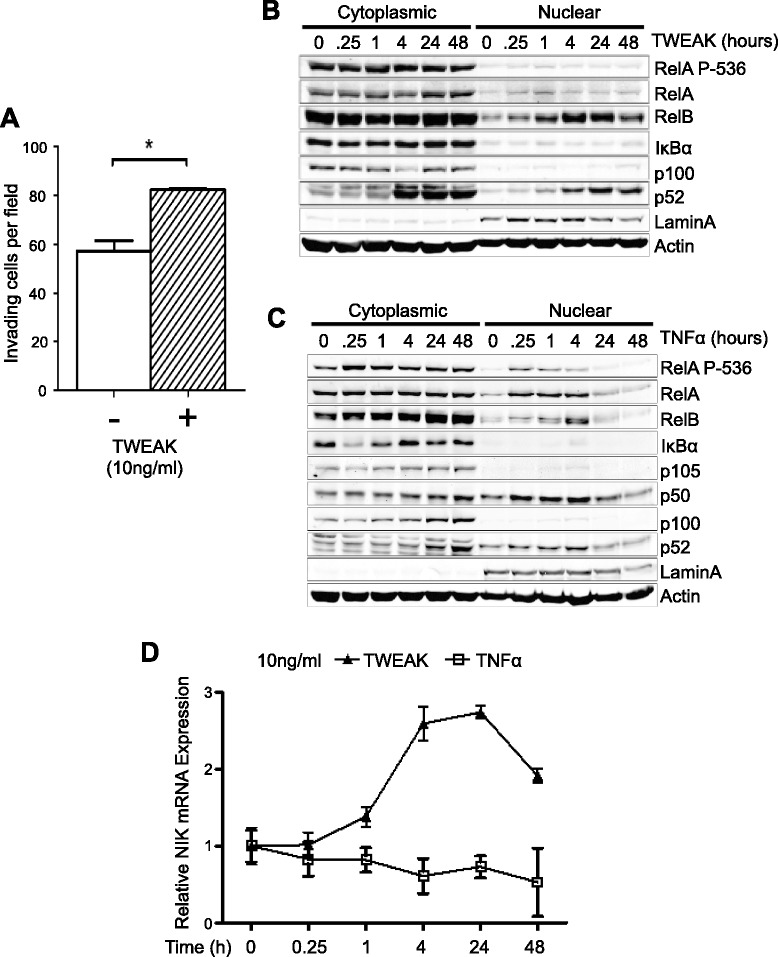
**TWEAK induces invasion, NIK mRNA expression, and noncanonical NF-κB nuclear translocation. (A)** Quantification of BT116 invasion density at 48 h with or without 10 ng/ml TWEAK in the collagen matrix. Data represent average numbers of invading cells per 1-mm^2^ field (n = 3 wells) ± S.E.M. *p < 0.05 relative to vehicle control using unpaired Student’s t-test. **(B)** Western blot analysis of proteins from cytoplasmic and nuclear fractions of BT116 cells stimulated with 10 ng/ml TWEAK for the indicated times using the indicated antibodies. **(C)** Western blot analysis of cytoplasmic and nuclear fractions as in Figure 3B post-stimulation with 10 ng/ml TNFα for the indicated times. **(D)** qRT-PCR analysis of NIK mRNA expression from BT116 cells. mRNA was collected at indicated time points after stimulation with TWEAK (10 ng/ml) or TNFα (10 ng/ml). Data represent average mRNA expression relative to RPLP0 mRNA (n = 3 replicates) ± S.D.

TWEAK has been reported to be an activator of both canonical and noncanonical NF-κB pathways [12,24,25]. However, very high concentrations of TWEAK (100 ng/ml) are required for activation of RelA [24]. We observed that treatment with a low, physiological concentration of TWEAK (10 ng/ml) was sufficient to increase glioma invasion in the 3-D collagen matrices (Figure 3A). Thus, we next examined the effect of TWEAK on NF-κB signaling. Glioma cells were treated with TWEAK (10 ng/ml) and western blot analysis was performed with cytoplasmic and nuclear protein lysates. We observed that TWEAK robustly induced noncanonical NF-κB activity, as evidenced by increased p100 processing to p52, as well as nuclear accumulation of both RelB and p52 (Figure 3B). Increased nuclear accumulation of RelB was observed as early as 15 minutes after treatment with TWEAK and reached peak levels at 4 hours, with a concurrent increase in p100 processing and nuclear p52 and sustained levels of both proteins after 48 hours (Figure 3B). Notably, nuclear translocation of RelA, RelA-P536, p105, p50, or c-Rel was not induced by TWEAK stimulation (Figure 3B and Additional file 1: Figure S2A). Moreover, no degradation of IκBα was observed in response to TWEAK (Figure 3B). In contrast, treatment with TNFα, a potent inducer of canonical NF-κB activity, led to rapid degradation of IκBα, and phosphorylation and nuclear translocation of RelA (Figure 3C), demonstrating that these cells have fully functional canonical NF-κB signaling. TNFα also induced RelB nuclear accumulation and p100 processing, albeit more transiently compared with TWEAK treatment (Figure 3C). As with TWEAK, TNFα induced RelB nuclear localization starting at 15 minutes, reaching maximal levels at 4 hours. However, levels of nuclear RelB returned to basal by 24–48 hours (Figure 3C). Similar results were observed in TNFα- and TWEAK-treated BT25 and U87 cells (data not shown). Finally, consistent with predominant activation of the noncanonical NF-κB pathway by TWEAK, we observed a robust increase in NIK mRNA levels as early as one hour after TWEAK treatment. TNFα, on the other hand, did not significantly alter NIK expression (Figure 3D). Induction of elevated NIK mRNA synthesis at 4 hours post-TWEAK also correlated with increased protein levels (Additional file 1: Figure S2B). Together, these data provide strong evidence supporting the ability of TWEAK to predominantly promote activation of the noncanonical NF-κB pathway in glioma.

### TWEAK-dependent regulation of MMP9 expression is mediated by the noncanonical NF-κB pathway

**Figure 4:**
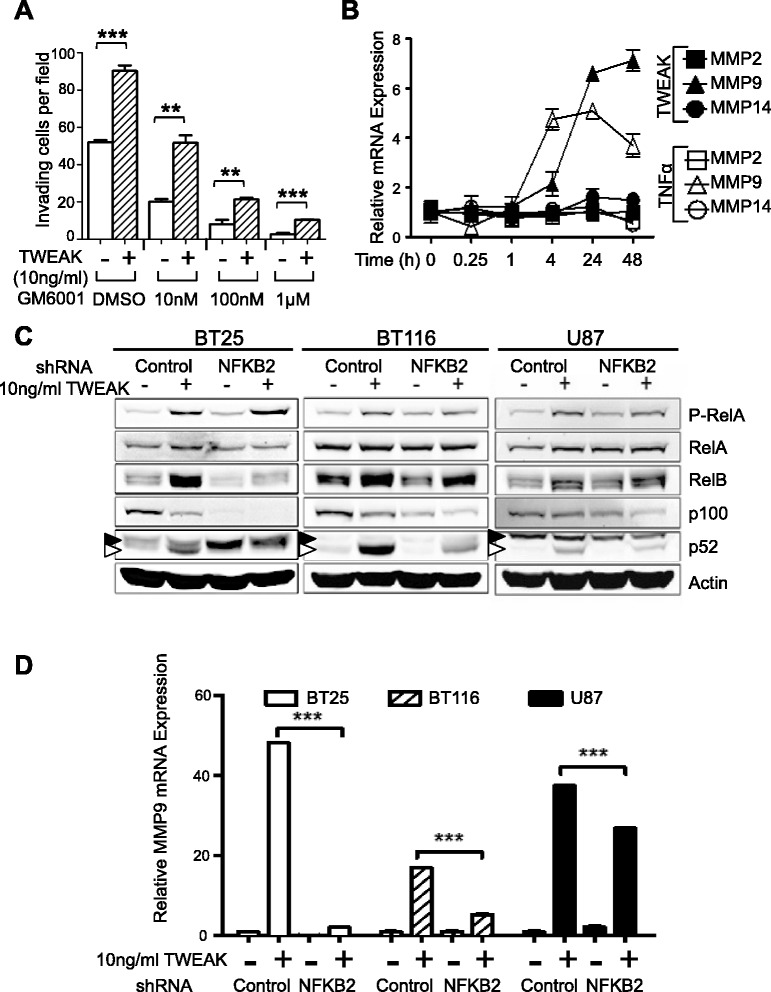
**MMPs are required for glioma invasion and TWEAK-induced MMP9 expression is p52-dependent. (A)** Quantification of invasion density at 48 h for BT116 cells with or without 10 ng/ml TWEAK in the collagen matrix. Cells were treated during the 48 h invasion assay with broad-spectrum MMP inhibitor GM6001 at indicated concentrations. Data represent average numbers of invading cells per 1-mm^2^ field (n = 3 wells) ± S.E.M. **p < 0.01 ***p < 0.001 between indicated groups using unpaired Student’s t-test. **(B)** qRT-PCR analysis of MMP mRNA expression in cultured BT116 cells. mRNA was collected at indicated time points after stimulation with TWEAK (10 ng/ml) or TNFα (10 ng/ml). Data represent average mRNA expression relative to GAPDH mRNA and normalized to untreated cells at t = 0 (n = 3 replicates) ± S.D. **(C)** Western blot of whole-cell lysates from cultured BT25, BT116, and U87 cells transduced with shRNA targeting p100/p52 (NFKB2) or a scrambled control (Control). Lysates were collected at 48 h after stimulation with 10 ng/ml TWEAK or vehicle control. White arrowhead indicates p52 band. Black arrowhead indicates non-specific band. **(D)** qRT-PCR analysis of MMP9 mRNA expression from cell lines in Figure 4C. mRNA was collected at 48 h after stimulation with 10 ng/ml TWEAK or vehicle control. Data represent average MMP9 mRNA expression relative to RPLP0 mRNA and normalized to untreated control (n = 3 replicates) ± S.D. ***p < 0.001 between indicated groups determined by unpaired Student’s t-test for each cell line.

Matrix Metalloproteinases (MMPs) plays key roles in many aspects of cancer pathogenesis, including regulation of extracellular matrix remodeling during invasion [16]. To investigate the role of MMPs in glioma cells invasion, we tested the effects of a pan MMP inhibitor, GM6001, in our 3-D invasion assays. We observed that GM6001 blocked invasiveness of untreated cells in a dose-dependent manner, with almost complete inhbition of invasion with 1μM (Figure 4A, Additional file 1: Figure S3A). The ability of TWEAK to promote invasion was also proportionately reduced with GM6001 treatment (Figure 4A). To examine the effect of TWEAK on expression of MMPs, we performed qRT-PCR on BT116 glioma cells treated with TWEAK (10 ng/ml) for 24 hours. Results from these experiments demonstrate that TWEAK robustly induced expression of MMP9 and negligibly affected expression of MMP2 and MMP14 (Figure 4B). Increased MMP9 expression was first discernable at 4 hours and peaking at 24–48 hours after TWEAK stimulation, concurrent with induction of RelB and p52 nuclear localization (see Figure 3B). TNFα also increased expression of MMP9 with more rapid kinetics but overall lower levels of induction compared to TWEAK (Figure 4B).

Given these findings, we surmised that the lower efficacy of GM6001 towards MMP9 activity (see Methods) might account for its inability to attenuate TWEAK-induced invasion. Therfore, to test the role of MMP9 in TWEAK-induced invasion we performed the 3-D invasion assay in the presence of a selective MMP9 inhibitor. MMP9 Inhibitor I did not have a significant effect on untreated U87 cells, however, the ability of TWEAK to promote invasion was significantly attenuated (Additional file 1: Figure S3B). Similar results were observed with BT114 and BT116 cells (data not shown).

To determine whether noncanonical NF-κB activity is required for TWEAK-induced MMP9 expression, we transduced BT25, BT116, and U87 cells with shRNAs targeting NFKB2 (p100) or a scrambled shRNA. Western blot analysis demonstrated that loss of NFKB2 resulted in a decrease in p100 protein levels and TWEAK-dependent processing to p52 (Figure 4C). TWEAK induction of RelB protein was also attenuated in NFKB2 knockdown cells (Figure 4C). qRT-PCR analyses demonstrated that although basal MMP9 mRNA levels were comparable between shNFKB2 and shControl lines, the ability of TWEAK to induce MMP9 expression was significantly impaired in NFKB2 knockdown cells (Figure 4D). These results demonstrate that TWEAK-mediated induction of NIK and noncanonical NF-κB pathway are critical for increasing expression of MMP9 and glioma cell invasion.

### Expression of NIK promotes gliomagenesis *in vivo*

**Figure 5:**
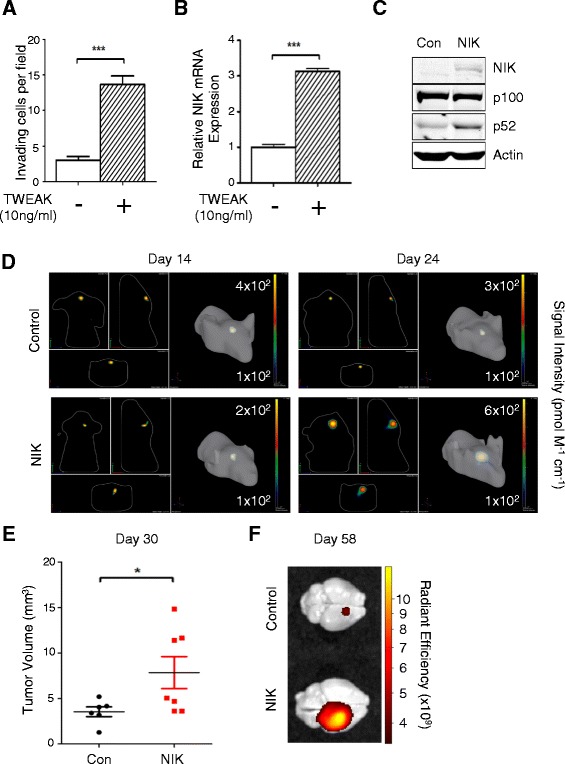
**NIK mRNA is up-regulated by TWEAK and NIK protein overexpression promotes tumorigenesis. (A)** Quantification of BT114 invasion density at 48 h with or without 10 ng/ml TWEAK in the collagen matrix. Data represent average numbers of invading cells per 1-mm^2^ field (n = 3 wells) ± S.E.M. ***p < 0.001 relative to vehicle control using unpaired Student’s t-test. **(B)** qRT-PCR analysis of NIK mRNA expression from BT114 cells. mRNA was collected at 48 h after stimulation with 10 ng/ml TWEAK or vehicle control. Data represent average NIK mRNA expression relative to RPLP0 mRNA and normalized to untreated control (n = 3 replicates) ± S.D. ***p < 0.001 relative to vehicle control using unpaired Student’s t-test. **(C)** Western blot analysis of BT114 glioma cells transduced with lentivirus containing NIK cDNA or luciferase cDNA (Con) using the indicated antibodies. **(D)** Representative IVIS images of 3-D rendering of tumors derived from DiD-stained intracranial-injected BT114-control (n = 7 mice) or BT114-NIK (n = 9 mice) cells at 14 and 24 days post-injection (n = 3 mice per group). Numbers indicate scale of Signal Intensity for each image. **(E)** Quantification of BT114 brain tumors in Figure 5D at 30 days. Data represent average tumor volume ± S.E.M. *p < 0.05 relative to BT114 control using unpaired Student’s t-test. **(F)** Representative IVIS *ex-vivo* images of DiD-stained BT114 brain tumors in Figure 5D at 58 days post-injection.

Our data thus far demonstrate that a low concentration of TWEAK promotes invasion and predominantly activates noncanonical NF-κB signaling in glioma tumor lines with high RelB expression. Notably, we observed that similar to BT116 cells, TWEAK was also able to promote invasion in BT114 glioma cells, which are minimally invasive and express low levels of RelB (Figure 5A; also see Figure 1). TWEAK also significantly increased levels of NIK mRNA in these cells (Figure 5B). Therefore, we investigated whether NIK could potentiate noncanonical NF-κB signaling and enhance gliomagenesis *in vivo* using an orthotopic mouse xenograft model. Ectopic expression of NIK in BT114 cells increased p100 processing to p52 compared with BT114 cells expressing vector control, indicating that the ectopically expressed NIK was functional (Figure 5C). RelA-P536, IκBα, p50, and c-Rel levels were unaffected by NIK overexpression, indicating that NIK did not significantly affect the canonical NF-κB pathway (Additional file 1: Figure S4). BT114-control and BT114-NIK cells were fluorescently labeled and injected into the right cortex of CD-1 nude mice. 3-D tomographic reconstruction of fluorescent images taken *in vivo* revealed that BT114-NIK cells formed larger and more dispersed tumors compared with BT114-control cells (Figure 5D). Quantification of tumor volume demonstrated that BT114-NIK cells formed significantly larger tumors (Figure 5E). Finally, *ex-vivo* images taken 58 days after intracranial injection confirmed that BT114-NIK cells formed significantly larger tumors compared with BT114-control (Figure 5E-F). Taken together, these data reveal a key role for NIK and noncanonical NF-κB signaling in glioma pathogenesis.

## Discussion

Aberrant activation of the canonical NF-κB pathway has been correlated with the promotion of brain cancer cell survival and invasion. However, roles for the noncanonical NF-κB pathway in CNS pathologies are less clear. RelB expression was previously described to be elevated in the aggressive mesenchymal glioma subtype [26] and we have recently identified a functional role for RelB as a key driver of mesenchymal gene expression and tumorigenesis [17]. A critical feature of the noncanonical NF-κB signaling pathway is its dependence on the regulation of NIK protein stability through proteasome-dependent degradation [7]. We show that a low concentration of TWEAK (10 ng/ml) preferentially and potently activates the noncanonical NF-κB signaling cascade, as evidenced by induction of p100 processing to p52, and nuclear accumulation of p52 and RelB (Figure 3). At this concentration, TWEAK failed to induce significant IκBα degradation and nuclear translocation of RelA (see Figure 3B), suggesting minimal TWEAK-induced activation of the canonical NF-κB pathway in glioma cells. Moreover, since a low concentration of TWEAK are sufficent to promote noncanonical NF-κB-mediated cell invasion, this pathway may be extremely important for initiating early, aggressive tumor dissemination.

Interestingly, we observed that TWEAK can promote noncanonical NF-κB signaling at the pre-translational level, as evidenced by accumulation of NIK mRNA in response to treatment with TWEAK. This induction of NIK mRNA was specific for TWEAK since TNFα, a potent inducer of the canonical NF-κB pathway, failed to increase NIK expression (see Figure 3D). Pre-translational regulation of NIK expression by TWEAK may be important for sustained activation of the noncanonical NF-κB pathway during the invasion process. TWEAK is synthesized in response to cellular injury and inflammation in tumor cells, as well as many cell types of the CNS, including neurons, microglia, and endothelial cells [27]. Therefore, TWEAK may function in both an autocrine and paracrine manner to robustly induce NIK expression, activate noncanonical NF-κB signaling and MMP expression, thereby promoting tumor cell invasion. Overall, these data provide compelling evidence that the pathological, pro-invasive effects of TWEAK are preferentially mediated by noncanonical NF-κB signaling.

Recently, noncanonical NF-κB signaling was shown to promote glioma invasion in response to non-toxic doses of BV-6, a potential chemotherapeutic agent that promotes cancer cell death through antagonism of cellular Inhibitors of Apoptosis proteins (c-IAPs) [28]. Our data demonstrate that both basal, constitutive noncanonical NF-κB activity, as well as induction of noncanonical NF-κB signaling by an endogenous cytokine, TWEAK, play critical roles in promoting glioma invasion. These findings demonstrate that noncanonical NF-κB signaling not only mediates therapy resistance, but also is important in normal tumor brain pathogenesis.

Lastly, it was recently reported that IKK-dependent, canonical NF-κB signaling suppresses NIK activity and noncanonical NF-κB signaling [29]. In the context of those findings, our data suggest that inhibition of canonical NF-κB pathway might result in increased constitutive noncanonical NF-κB activity, therey promoting tumor cell invasion and pathogenesis. Notably, the ability of TWEAK to induce NIK expression and promote invasion is not only observed in glioma cells expressing high levels of RelB, but also in glioma cells with low levels of endogenous RelB expression (Figure 5). Altogether, these data suggest that blocking TWEAK signaling and noncanonical NF-κB activation, alone or in combination with inhibition of canonical NF-κB signaling, will be more efficacious for attenuation of tumor cell invasion and, therefore, have therapeutic value in a broad range of glioma subtypes.

## Conclusion

Here, we establish a key role for noncanonical NF-κB signaling in glioma that is independent of the canonical NF-κB pathway. Specifically, we demonstrate that RelB promotes glioma invasion in the absence of RelA, and that TWEAK preferentially regulates noncanonical NF-κB signaling in glioma through a novel, signal-specific induction of NIK expression. Morevoer, NIK is sufficient to promote brain tumor growth in vivo. To date, therapeutic strategies targeting NF-κB have mostly focused almost exclusively on inhibition of the canonical NF-κB pathway [30]. Our findings provide a compelling rationale for considering TWEAK/Fn14 and NIK inhibition as therapeutic strategies for aggressive glioma, as well as other highly invasive cancers.

## Methods

### Cell culture & reagents

BT lines from glioma patients (BT25, BT114, BT116, BT132) were obtained as described previously [31]. 293 T, U87-MG, and U373 cells were purchased from ATCC. Glioma cell lines were cultured in DMEM/F12 + 10%FBS (1X Glutamax, 1X Pen/Strep) or Neural Stem Cell (NSC) medium (DMEM/F12, 1X B-27 Supplement minus Vitamin A, 1X Glutamax, 50 ng/ml EGF, 50 ng/ml bFGF, 1X Pen/Strep). All cell culture reagents were from Life Technologies (Grand Island, NY). rhTNFα was obtained from Promega (Madison, WI), and rhTWEAK was obtained from PeproTech (Rocky Hill, NJ). GM6001 inhibitor and MMP Inhibitor I were obtained from EMD Millipore (Temecula, CA).

### 3-D invasion assays

Invasion assays were performed as described previously [17,18]. In brief, Type I collagen extracted from rat tail tendons [32] was diluted to 2 mg/ml in DMEM/F12 (1× Pen/Strep) and matrices polymerized in 96-well plates. 40,000 cells per well were seeded in 100 μl DMEM/F12 (1× Pen/Strep, 1× Glutamax) without growth factors or serum. Cells were fixed with 3% glutaraldehyde solution after 48 h of invasion and stained with 0.1% toluidine blue. Invasion density was quantified by counting cells below the plane of the monolayer by bright-field light microscopy using a 10 × 10 ocular grid at 10× magnification corresponding to a 1 mm^2^ field. Numbers in equivalent fields were counted (n = 3 wells). Cross-sectional images were taken with an Olympus CKX41 inverted microscope and Q-Color 3 camera.

### Western blots

Whole-cell lysates were prepared using RIPA buffer with 1X Halt protease and phosphatase inhibitors (Thermo Scientific, Rockford, IL). Cytoplasmic and nuclear extracts were prepared as previously described [33]. 25-40 μg of protein was separated in replicate 9% polyacrylamide (29:1 Bis) Tris gels and transferred to replicate nitrocellulose membranes for probing proteins in parallel. After transfer, gels were stained with GelCode Coomassie (Thermo Scientific, Rockford, IL) and scanned with the IR700 channel of an Odyssey Infrared Imaging system (LI-COR Biosciences, Lincoln, NE) for first verification of even protein loading. Westerns were performed by blocking membranes in 1:1 PBS/Odyssey Blocking Buffer (LI-COR Biosciences, Lincoln, NE) prior to co-incubation with mouse and rabbit primary antibodies. Simultaneous detection was performed using goat anti-rabbit IRDye800CW and goat anti-mouse IRDye680 secondary antibodies (LI-COR Biosciences, Lincoln NE). Membranes were probed in parallel for simultaneous detection of multiple antibodies in replicate membranes without stripping. Western blots and Coomassie-stained gels were scanned with the Odyssey Imager. Color images were scanned with a LI-COR Infrared Imaging System, converted to black and white, and analyzed using the LI-COR Image Studio software. Quantitative analysis of indicated images was performed using Image Studio or Image J software (NIH, Bethesda, MA). The following Santa Cruz Biotechnology (SC, Santa Cruz, CA) and Cell-Signaling Technology (CST, Danvers, MA) antibodies were used: RelB (CST-4922), RelA (SC- 8008), Phospho-RelA Ser536 (CST-3033), c-Rel (CST-4727), p100/p52 (CST-3017), p105/p50 (CST 13681), LaminA (SC 56137), IkBα (CST 4814), NIK (CST 4994), β-Actin (SC-69879).

### Plasmids

pLenti6 overexpression constructs for RelB and NIK were generated by subcloning cDNA into pLenti6-V5-DEST (Addgene, Cambridge, MA) using the GATEWAY™ Cloning System. Luciferase (Promega, Madison, WI) or tagRFP (Evrogen, Moscow, Russia) coding sequences were subcloned into pLeni6-V5-DEST and used as controls for RelB and NIK overexpression. Mission™ Lentiviral shRNA plasmids for RelA, RelB and control were purchased from Sigma-Aldrich (St. Louis, MO). shRelB, as well as its control, was described previously as shRelB-3 [17]. shNFKB2 and shControl oligonucleotides (Integrated DNA Technologies, Coralville, IA) were subcloned into pLKO.1-puro (Addgene, Cambridge, MA). Exact sequences are available upon request.

### Lentivirus production and transduction

293T cells were transfected with 7 μg of lentiviral plasmids using 21 μg of polyethyleneimine (Polysciences Inc., Warrington, PA). Lentiviruses were harvested after 3 days and used to infect 2x10^5^ glioma cells. Transduced cells were selected for 72 h in DMEM/F12 NSC medium containing 0.6 μg/ml Puromycin or 3 μg/ml Blasticidin (Invivogen, San Diego, CA) to verify stable transduction. Cells were continuously selected during culture with 0.6 μg/ml Puromycin (shRNA constructs) and/or 6 μg/ml Blasticidin (overexpression constructs).

### Quantitative reverse-transcriptase PCR

Total RNAs were isolated from cells using Purelink™ RNA Mini Kit (Life Technologies, Carlsbad, CA). cDNA was synthesized from 1 μg of total RNA using SuperScript® III Reverse Transcriptase (Life Technologies, Carlsbad, CA) following manufacturer’s instructions. Quantitative RT-PCR was performed using SYBR® Green PCR Master Mix (Applied Biosystems, Foster City CA). Expression of mRNA was normalized to either GAPDH or RPLP0 expression levels. The following primers were used in amplifications: GAPDH 5′-AATGAAGGGGTCATTGATGG-3′, 5′-AAGGTGAAGGTCGGAGTCAA-3′; RPLP0 5′- TCGTCTTTAAACCCCTGCGTG-3′, 5′-TGTCTGCTCCCACAATGAAAC-3′; MMP-2 5′-AAGAAGTAGCTGTGACCGCC-3′ 5′-TTGCTGGAGACAAATTCTGG-3′; MMP9 5′-GCACTGCAGGATGTCATAGG-3′ 5′-ACGACGTCTTCCAGTACCGA-3′; MMP-14 5′-TGCCTACCGACAAGATTGATG-3′ 5′-ATCCCTTCCCAGACTTTGATG-3′; NIK 5′-TTCAGCCCCACCTTTTCAG-3′ 5′-ACGCTTTCCCTTCCAACAC-3′. All experiments were performed at least three times with three replicates per sample.

### Orthotopic mouse xenografts

All animal experiments were done in compliance with IACUC, AAALAC and Texas A&M University Health Science Center Biosafety guidelines using an IACUC-approved Animal Use Protocol (# 2012–174). For orthotopic tumor inoculations, cells were labeled using a DiD (DiIC18(5); 1,1′-dioctadecyl-3,3,3′,3′- tetramethylindodicarbocyanine, 4-chlorobenzenesulfonate salt) cytoplasmic membrane dye; abs/em = 644/655 (Biotium, Hayward, CA) according to the manufacturers directions. 0.5-1×10^6^ cells in 3–5 μl phosphate buffered saline were injected into the right cortex of 4–6 week old CD-1 nude mice (n = 3 each). Tumor cells were imaged *in vivo* at indicated times post-injection using an IVIS Spectrum In Vivo Imaging System and Living Image Software (Perkin Elmer, Waltham, MA). Tumor volume was calculated from the formula: Volume = 0.5 × length × width^2^. The highest and lowest numbers for calculated tumor volume in each group were excluded from analysis.

### Statistical analyses

GraphPad Prism 5 software was used for all statistical analyses. Paired student’s *t*-test or one-way analysis of variance (ANOVA) with Tukey’s honest significant difference (H.S.D.) post-test was performed and an α-value of 0.05 was used as criteria for statistical significance.

## Additional file

Additional file 1: Figure S1. Efficiency of RelA knockdown among multiple shRNA constructs and quantification of RelB overexpression. **Figure S2.** TWEAK specifically induces noncanonical NF-κB activation in glioma cells via NIK protein accumulation. **Figure S3.** TWEAK-enhanced invasion is attenuated by broadspectrum MMP and MMP9-selective inhibition. **Figure S4.** NIK overexpression specifically induces p100 processing in glioma cells.

## Abbreviations

3-D: Three-dimensional
BAFFR: B cell activating factor receptor
Fn14/TNFRSF12A: Fibroblast growth-factor-inducible Immediate-early Response Protein 14/Tumor necrosis factor receptor superfamily member 12A
IKK: Inhibitor of kappa B kinase
IκB: Inhibitor of kappa B
MMP: Matrix metalloproteinase
NF-κB: Nuclear factor kappa B
NFKB2: Nuclear factor of kappa light polypeptide gene enhancer in b-cells 2
NIK: NF-κB-inducing kinase
TNF: Tumor necrosis factor
TRAF: TNF receptor associated factor
TWEAK: Tumor necrosis factor-like weak inducer of apoptosis
