# Supplementary figures and images for: Tumor necrosis factor-like weak inducer of apoptosis (TWEAK) promotes glioma cell invasion through induction of NF-κB-inducing kinase (NIK) and noncanonical NF-κB signaling
Source: Mol Cancer. 2015 Jan 27;14(1):9. doi: 10.1186/s12943-014-0273-1 (PMC4320546; doi:10.1186/s12943-014-0273-1)

Supplemental Figure 3

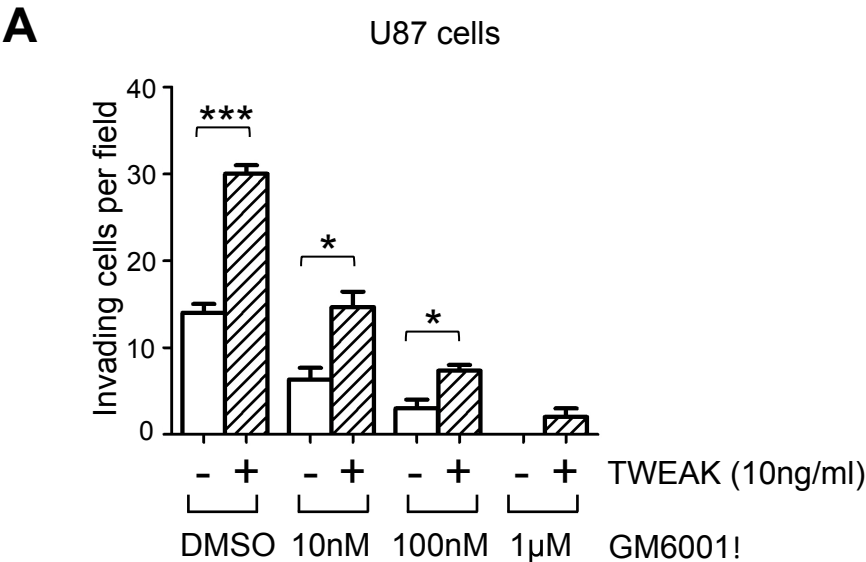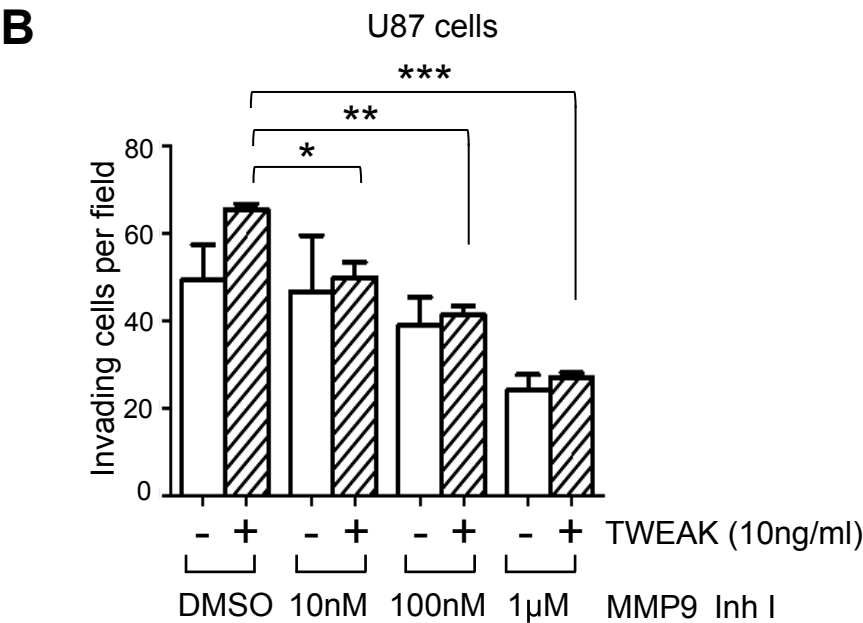

Supplement: Additional file 1: Figure S1. — Efficiency of RelA knockdown among multiple shRNA constructs and quantification of RelB overexpression. Figure S2. TWEAK specifically induces noncanonical NF-κB activation in glioma cells via NIK protein accumulation. Figure S3. TWEAK-enhanced invasion is attenuated by broadspectrum MMP and MMP9-selective inhibition. Figure S4. NIK overexpression specifically induces p100 processing in glioma cells. [file 12943_2014_273_MOESM1_ESM.pdf]
